# Effect of Wing-Wing Interaction on the Propulsive Performance of Two Flapping Wings at Biplane Configuration

**DOI:** 10.1155/2018/8901067

**Published:** 2018-09-20

**Authors:** Jianyang Zhu, Bin Lei

**Affiliations:** ^1^School of Machinery and Automation, Wuhan University of Science and Technology, Wuhan 430081, China; ^2^Hubei Key Laboratory of Mechanical Transmission and Manufacturing Engineering, Wuhan University of Science and Technology, China

## Abstract

The biplane counter-flapping wing is a special type of wing flapping which is inspired from the fish and insect in nature. The propulsive performance is one of the most important considerations for this kind of flapping wing. This paper is aimed at providing a systematic synthesis on the propulsive characteristics of two flapping wings at biplane configuration based on the numerical analysis approach. Firstly, parameters of this special flapping wing are presented. Secondly, the numerical method for simultaneously solving the incompressible flow and counter-flapping motion of the wing is illustrated, and the method is then validated. Thirdly, the effects of phase angle and mean wing spacing on the propulsive characteristics of the biplane counter-flapping wing are analyzed. Finally, the quantification effects of the phase angle and mean wing spacing on the propulsive characteristics of the biplane counter-flapping wing can be obtained. The analysis results in this study will provide useful guidelines to design an effectively propulsive system applying for the flapping micro air or underwater vehicle.

## 1. Introduction

The aerodynamic performance of two flapping wings at biplane configuration has gained popularity investigating in recent years due to their potential to apply for propulsion of flapping micro air and underwater vehicle [1–4]. The propulsive performance is the one of the most important considerations for successfully designing this kind or type of flapping vehicle. Therefore, many studies have been carried out to focus on optimal propulsive performance of these kinds of wings, and considerable conclusions have been achieved [5–8].

Tuncer and Kaya [9] numerically studied the unsteady, viscous flow around an oscillating biplane airfoil by solving 2-D unsteady Reynolds-averaged Navier-Stokes (URANS) equations. They concluded that the oscillating biplane airfoil can produce 20–40% more thrust than a single airfoil. However, the propulsive efficiency was not considered in their study. Later, in their other study [10], the parameters of the oscillating biplane airfoil were optimized for best propulsive performance. The mean distance between two airfoils was fixed at 1.4 chord length, and only thrust-generating performance of the airfoil was considered. It was found that the flapping airfoils in a biplane configuration can produce as much as 25% more thrust per airfoil than a single flapping airfoil at the Strouhal number range of 0.17–0.25.

Deng et al. [11] conducted a numerical study to investigate the propulsive performance of the biplane counter-flapping flexible wings. The flapping motion of the wings was described by a sinusoidal plunge and defined chordwise deformation motion. The mean distance between two wings was also fixed but at a value of 0.4 chord length in their study. The results revealed that the biplane wing with counter-flapping configuration has both a larger thrust and propulsive efficiency in comparison to a single flapping airfoil. While, recently, Shen and Cai [12] through analyzing aerodynamic characteristics of three different flapping configurations: single wing, tandem wings, and biplane wings, indicated that biplane wing model has the most inefficient propulsive performance, however, it can provide the largest thrust, and the tandem arrangement is the most efficient configuration.

It can be summarized from the above studies that there is still no consensus on whether the biplane flapping wing can both achieve high thrust and efficiency, and phase angle as well as the mean wing spacing affects the aerodynamic characteristics of biplane wings. In addition, almost all numerical studies assumed a laminar flow even when the Reynolds number of the wing is as high as 10^4^, which is different from the real working fluid around the wing. To fill in these gaps, we perform a numerical study to systematically investigate the effect of phase angle as well as the mean wing spacing on the aerodynamics of flapping wings in biplane configuration. A 2D biplane counter-flapping wing under forward flight is studied, where the N-S solver for fluid flow around the wing coupled with dynamic mesh technology for the wing motion is solved, so that the fluid, wing and wing interaction can be taken into account properly. The flow field, thrust forces, and propulsive efficiency of the wing are examined in detail.

## 2. Parameters of Flapping Biplane Wing

### 2.1. Flapping Kinematics

**Figure 1:**
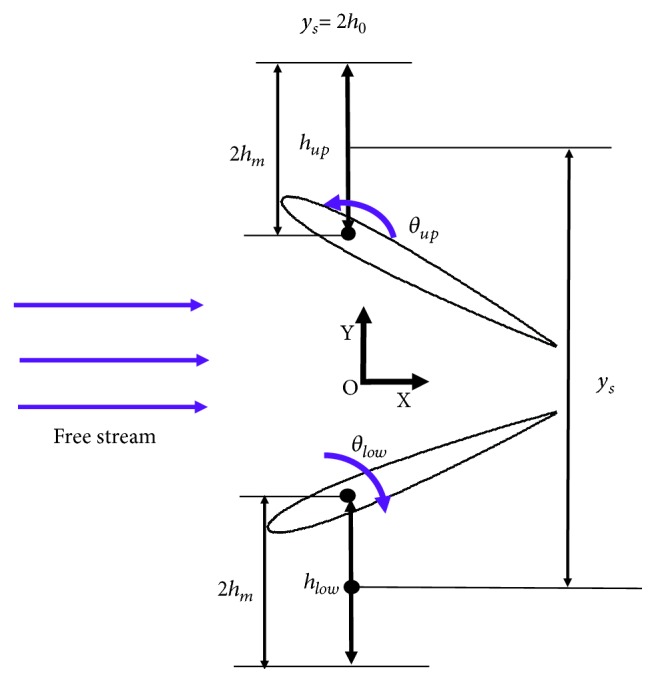
The schematic of the biplane counter-flapping wing.

A 2D biplane counter-flapping wing oscillating harmonically in plunging and pitching as shown in Figure 1 is considered in this paper. NACA0014 airfoil is employed to represent the cross section of a wing. The up and low wings perform a mirroring flapping with respect to the horizontal axis *X*. The equations of the plunging and pitching motions of the up and low wings are defined as follows:

(1) $$\begin{array}{c} h_{up}=h_{0}-h_{m}\cos\left(2\pi \text{ft}\right),\\ \theta_{up}=-\theta_{m}\cos\left(2\pi \text{ft}+\phi \right),\\ h_{low}=-h_{0}+h_{m}\cos\left(2\pi \text{ft}\right),\\ \theta_{low}=\theta_{m}\cos\left(2\pi \text{ft}+\phi \right), \end{array}$$

where *h*_0_ is the initial plunging position, *h*_*m*_ is the plunging amplitude, *f* is the flapping frequency, *θ*_*m*_ is the pitching amplitude, and *ϕ* is the phase angle difference between plunging and pitching. Note that the pitching axis is located at 0.25 chord length from the leading edge. The mean wing spacing between up and low wings is defined as follows:

(2) $$\begin{array}{c} y_{s}=2h_{0}. \end{array}$$

### 2.2. Aerodynamic Parameter Definition

For the biplane counter-flapping wing, the Reynolds number and reduced frequency are defined as follows:

(3) Re=U∞cν,k=2πfcU∞,

where *U*_∞_ is the free stream velocity, *ν* is the air kinematic viscosity, and *c* is the chord length of the wing.

The lift and thrust coefficients of the wing are described as follows:

(4) $$\begin{array}{c} C_{L}=\frac{F_{Y}}{0.5\rho U_{\infty }^{2}c},\\ C_{T}=\frac{-F_{X}}{0.5\rho U_{\infty }^{2}c}, \end{array}$$

where *F*_*Y*_ is the lift (along *Y* direction as shown in Figure 1), *F*_*X*_ is the drag force (along *X* direction as shown in Figure 1), and *ρ* is the fluid density.

There are two parts of the energy consumption of the wing oscillating in plunging and pitching, one part is to support the plunging motion and the other part is to support the pitching motion. Therefore, the energy consumption of the wing can be defined as follows:

(5) $$\begin{array}{c} P=-F_{Y}\dot{h}-M\dot{\theta }, \end{array}$$

where *M* is the pitching moment of the wing, $\dot{h}$ is the plunging velocity of the wing, and $\dot{\theta }$ is the pitching velocity of the wing. The energy coefficient of the wing is defined as follows:

(6) $$\begin{array}{c} C_{P}=\frac{-F_{Y}\dot{h}-M\dot{\theta }}{0.5\rho U_{\infty }^{3}c}. \end{array}$$

Due to the biplane counter-flapping wing, the instantaneous lift of the summation of the up and down wings is closed to zero; therefore, only the propulsive efficiency is considered which can be calculated as follows:

(7) $$\begin{array}{c} \eta_{T}=\frac{\bar{C_{T}}}{\bar{C_{P}}}, \end{array}$$

where $\bar{C_{T}}$ and $\bar{C_{P}}$ are the mean values of thrust coefficient and energy coefficient, respectively.

## 3. Numerical Method

### 3.1. Fluid Solver

Based on the Reynolds number (at range of 10^4^–10^5^) of larger insect and bird flights, it is assumed that the flow around the biplane counter-flapping wing is incompressible, with constant viscosity and turbulence. The governing equations can be given as follows:

(8) $$\begin{array}{c} \begin{array}{c} \frac{\partial u_{i}}{\partial x_{i}}=0, \end{array}\\ \rho \frac{{Du}_{i}}{Dt}=-\frac{\partial p}{\partial x_{i}}+\frac{\partial }{x_{j}}\left[\mu \left(\frac{\partial u_{i}}{\partial x_{j}}+\frac{\partial u_{j}}{\partial x_{i}}\right)\right]+\frac{\partial }{\partial x_{j}}\left(-\rho \bar{u_{i}^{\prime }u_{j}^{\prime }}\right),\\ \rho \bar{u_{i}^{\prime }u_{j}^{\prime }}=\frac{2}{3}\rho k_{v}\delta_{ij}-\mu_{t}\left(\frac{\partial u_{i}}{\partial x_{j}}+\frac{\partial u_{j}}{\partial x_{i}}\right), \end{array}$$

where *i* and *j* are the subscripts of the velocity, *u*_*i*_ and *u*_*j*_ are the velocity vector, *p* is the pressure, *μ* is the fluid dynamic viscosity, $-\rho \bar{u_{i}^{\prime }u_{j}^{\prime }}$ is the Reynolds stress, *δ*_*ij*_ is the Kronecker function, *k*_*v*_ is the turbulent kinetic energy, and *μ*_*t*_ is the turbulent viscosity.

In order to solve the governing (8), Spalart-Allmaras turbulence model which is suggested for low Reynolds number turbulence flow simulation [13, 14] is employed to solve the Reynolds stress. The second-order upwind algorithm is employed for space discretization and first-order implicit algorithm for time discretization, the coupling between the pressure and the velocity is achieved by means of the SIMPLEC algorithm, and the counter-flapping wing is realized and controlled by dynamic mesh technology.

### 3.2. Mesh Generation and Boundary Conditions

**Figure 2:**
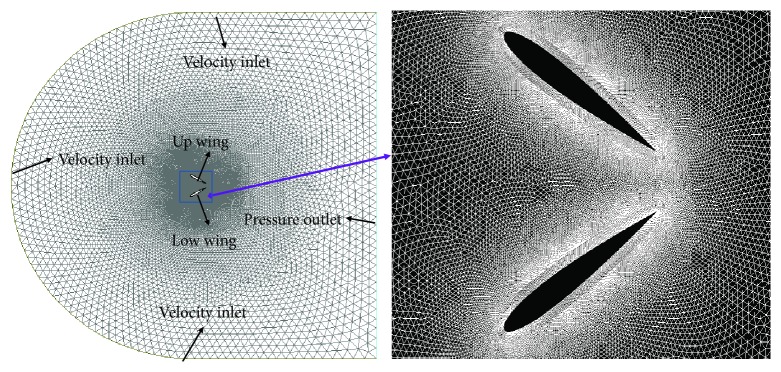
Mesh system of the biplane counter-flapping wing.

A hybrid mesh system is employed where a C-type computational domain (shown in Figure 2) containing an inner domain and an outer domain is used. Ten rows of boundary layer are used to encompass the up and low wings in the inner domain which move according to the wing kinematics, and the first grid is located at 0.003*c* from the wing surface; the growth rate of the grid is 1.05. Triangular cells are used in the outer field where remeshing takes place at each time step. Note that in the following simulation, all the *y*+ values on the wing surfaces are less than 15, which have been proved to be accurate for simulating the flow with Spalart-Allmaras turbulence model by Salim and Cheah [15].

No slip wall boundary condition is applied on the surface of the counter-flapping wing. The incoming fluid flows from left to right; velocity inlet boundary condition is applied at the left, top, and bottom boundaries which can be described as follows:

(9) $$\begin{array}{c} u=U_{\infty },\\ v=0, \end{array}$$

and pressure outlet applied at the left boundary can be described as follows:

(10) $$\begin{array}{c} p=P_{\infty }, \end{array}$$

where *P*_∞_ is the standard atmospheric pressure.

### 3.3. Grid Size and Iteration Time Step Validation

**Figure 3:**
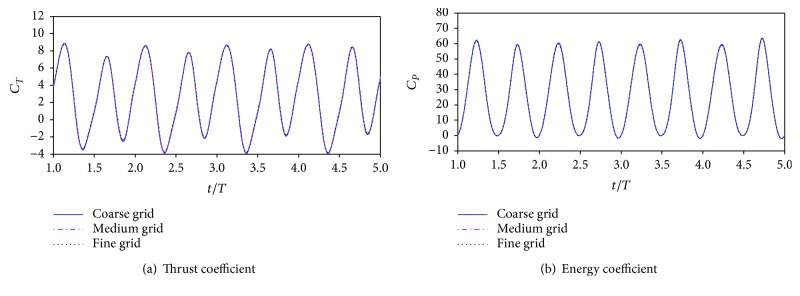
The time variation of thrust and energy coefficient with three different grid systems.

Grid and time step independence studies are carried out with three grids and three time step variations per flapping cycle tested for the same biplane counter-flapping wing case. The parameters of the simulation case are the chord wise of the wing *c* = 0.1 m, *h*_0_ = 0.75*c*, *h*_*m*_ = 0.25*c*, *θ*_*m*_ = 30°, *k* = 4.30, *ϕ* = 0°, and Re = 1 × 10^4^. The coarse grid contains 43,358 triangular cells and 2540 quadrilateral cells; the first grid is located at 0.006*c* from the wing surface. The medium contains 60,494 triangular cells and 8160 quadrilateral cells; the first grid is located at 0.003*c* from the wing surface. The fine contains 88,256 triangular cells and 19,584 quadrilateral cells; the first grid is located at 0.00015*c* from the wing surface. The iteration time step is fixed at *T*/2000 (*T* = 1/*f*) for the grid size validation. Since the instantaneous lift force of the system is close to zero due to the biplane counter-flapping wing, only the comparisons of thrust and energy coefficients at different resolutions are given. The results in Figure 3 show that the differences of thrust and energy coefficients of the three grid schemes are very small (not more than 1%). However, medium grid is employed for the next simulations.

**Figure 4:**
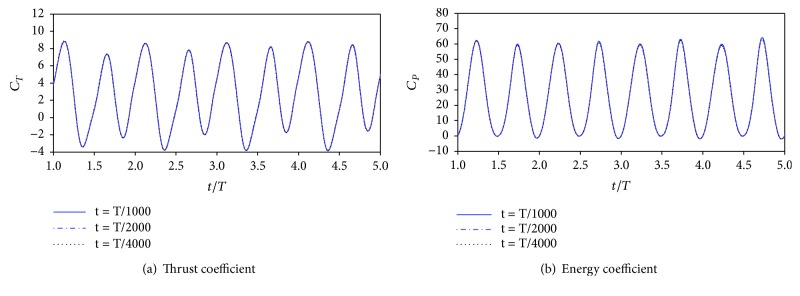
The time variation of thrust and energy coefficients with three different iteration time steps.

To validate iteration time step independence, another two iteration time steps (*T*/1000 and *T*/4000) are considered. It is seen from Figure 4 that the differences of thrust and energy coefficients of the three iteration time step scheme are very small (not more than 1%). However, *T*/2000 is employed for the next simulations.

### 3.4. Solving Validation

**Figure 5:**
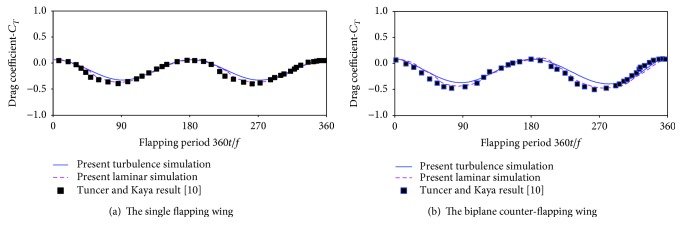
Time variation of drag coefficients of the flapping wing at Re = 1 × 10^4^.

To validate the numerical method used in this work which is robust and effective to simulate the interaction of flapping wing and fluid flow, two typical flapping wings which were numerically studied by Kaya et al. [10] are studied. The simulation parameters of the single flapping wing are *h*_*m*_ = 0.54*c*, *θ*_*m*_ = 9.93°, *k* = 1.0, *ϕ* = 84.3°, and Re = 1 × 10^4^, and the parameters of the biplane counter-flapping wing are *h*_*m*_ = 0.54*c*, *θ*_*m*_ = 10.4°, *k* = 1.0, *ϕ* = 79.9°, and Re = 1 × 10^4^. Since in [10], the flow around wing was assumed laminar at Re = 1 × 10^4^, and NACA0012 was employed to represent the wing shape. In this section, in order to compare the results with reference [10], the same wing shape (NACA0012) is employed, and both the laminar and turbulence flow around wing are simulated. The resulting drag coefficient (−*C*_*T*_) of the wing is plotted against flapping period (360 *t*/*f*) as shown in Figure 5, where the result by Kaya et al. [10] is also presented for comparison. It is seen from this figure that the present result with laminar simulation has larger negative drag coefficient amplitude than the result with turbulence simulation, which leads both the single and biplane counter-flapping wings under laminar flow generating larger thrust. On the other hand, the time variation of drag coefficient of the present result with laminar simulation and literature's data is in good agreement. All in all, the present numerical simulation can grasp the main features of the flow around flapping wing.

## 4. Results and Discussion

To study the effect of wing-wing interaction on the propulsive performance of the 2D biplane counter-flapping wing under forward flight, the results of the 2D biplane counter-flapping wing are needed to compare to one with a single flapping up wing. The biplane counter-flapping wing is performed with *c* = 0.1 m, *h*_*m*_ = 0.25*c*, *θ*_*m*_ = 30°, *k* = 4.30, and Re = 1 × 10^4^, and the parameters of the biplane counter-flapping wing are characterized by the mean value of the up and low wings for comparison. The details of phase angle as well as the mean wing spacing on the thrust and propulsive efficiency of the wing will be analyzed in the following, and results for all cases were taken after periodic thrust had been established which required at least five flapping cycle simulations.

### 4.1. Phase Angle Influence Study

To study the effect of phase angle *ϕ* on the propulsive performance of the biplane counter-flapping wing, we fix the mean wing spacing *y*_*s*_ = 1.5*c*, and the biplane counter-flapping wing with *ϕ* = −180° to 180° with interval of 30° is considered.

**Figure 6:**
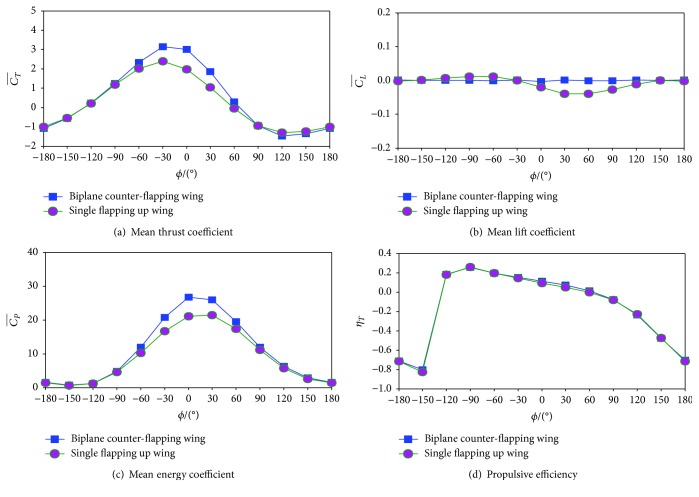
The performance of the biplane counter-flapping wing and single flapping up wing with different phase angle *ϕ*.

Figure 6 shows the performance of the biplane counter-flapping wing with different phase angle *ϕ*. Note that the results of the single flapping up wing are also presented for comparison.

It is seen from Figure 6(a) that similar variation trend of the mean thrust coefficient of the biplane counter-flapping and single flapping up wing with phase angle is observed, and the maximum mean thrust coefficient is achieved when the wing with *ϕ* = −30°, which is different from the result obtained by Tuncer and Kaya [9] that the frequency angle is at −120° for *k* = 0.5 and *θ*_*m*_ = 10° and at −60° for *k* = 1.0 and *θ*_*m*_ = 10°. This difference can be explained by the larger reduced frequency *k* employed in this study. It is also found in this figure that there exists an optimal phase angle range (*ϕ* = [−90°, 60°]) where the biplane counter-flapping wing produces 5.60%–77.14% more thrust than the single flapping up wing and the maximum increasing is achieved at *ϕ* = 0°. These results indicate that the biplane counter-flapping wing has better thrust-generating performance than the single flapping wing.

It is seen from Figure 6(b) that for biplane counter-flapping and single flapping up wings, the mean lift coefficient of the wing is all close to zero; however, the single flapping up wing has larger amplitude than the biplane counter-flapping wing.

It is seen from Figure 6(c) that the same as mean thrust coefficient, similar variation trend of the mean energy coefficient of the biplane counter-flapping and single flapping up wings with phase angle is observed; however, the maximum energy coefficient is achieved when the wing with *ϕ* = 0°.

It is seen from Figure 6(d) that the power efficiency of the biplane counter-flapping and single flapping up wings has similar variation with phase angle, different from the mean thrust coefficient; the maximum power efficiency is achieved when the wing with *ϕ* = −90°. It is also found that there exists an optimal phase angle range (*ϕ* = [−30°, 60°]) where the biplane counter-flapping wing has 6.81%–45.96% more efficiency than the single flapping up wing, and the maximum is achieved at *ϕ* = 30°, which indicates that the biplane configure wing has better propulsive efficiency performance than the single flapping wing.

It must be emphasized here that the maximum mean thrust coefficient increasing (77.14%) is achieved when the phase angle is *ϕ* = 0°, while the maximum propulsive efficiency increasing (45.96%) is achieved when the phase angle is *ϕ* = 30°. However, according to the study of Kaya et al. [10] for a biplane counter-flapping wing with smaller pitch amplitude and reduced frequency (*θ*_*m*_ = 10° and *k* = 1.00) under laminar simulation, the optimal phase angle of the wing for thrust production is 60°. This is different from what is obtained in the present study where the wing with larger pitch amplitude and reduced frequency (*θ*_*m*_ = 30° and *k* = 4.30) under turbulence simulation is considered.

To analyze the mechanism of how the wing-wing interaction enhances the propulsive performance of the flapping wing details, a typically biplane counter-flapping wing case with phase angle *ϕ* = 0° which has both larger mean thrust coefficient and propulsive efficiency than the single flapping up wing as shown in Figure 6 is studied in detail, and the result for the single flapping up wing is also presented for comparison.

**Figure 7:**
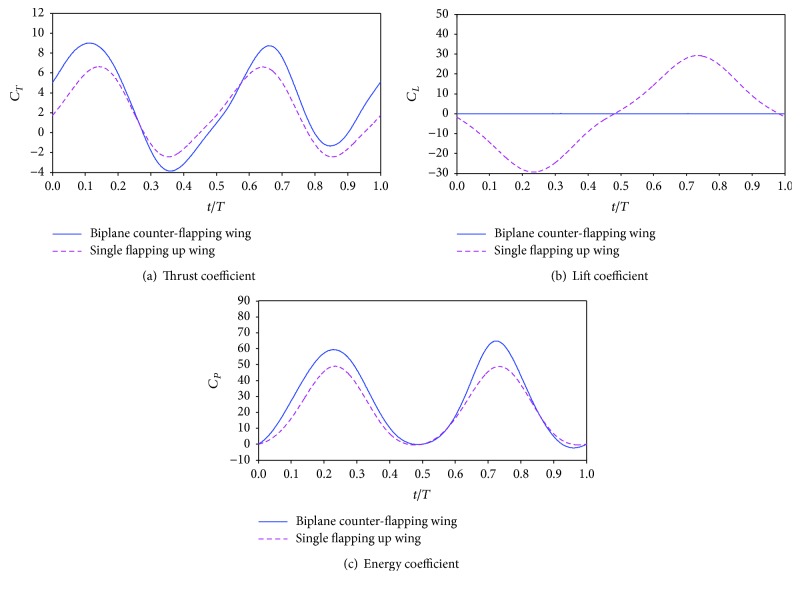
Time variation of the thrust coefficient, lift coefficient, and energy coefficient of the biplane counter-flapping wing and single flapping up wing with *ϕ* = 0°.

Figure 7 plots the time variation of thrust coefficient, lift coefficient, and energy coefficient of the two considered wings. It is obvious in Figure 7(a) that the biplane counter-flapping wing has larger thrust coefficient than the single flapping up wing almost during the whole flapping cycle, expect at *t* = 0.25 *T*–0.58 *T*, which results the biplane counter-flapping wing to have larger mean thrust coefficient as shown in Figure 6(a). It is seen from Figure 7(b) that zero lift coefficient is observed during the whole flapping cycle for the biplane counter-flapping wing, which may be beneficial for aerodynamic stability of the wing; however, for the single flapping up wing, the periodic time variation of lift coefficient is observed. As shown in Figure 7(c), the energy coefficient of the biplane counter-flapping wing has larger amplitude than the single flapping up wing almost during the whole flapping cycle, expect at *t* = 0.90 *T*–1.00 *T*, which results the biplane counter-flapping wing to have larger mean energy coefficient as shown in Figure 6(c).

**Figure 8:**
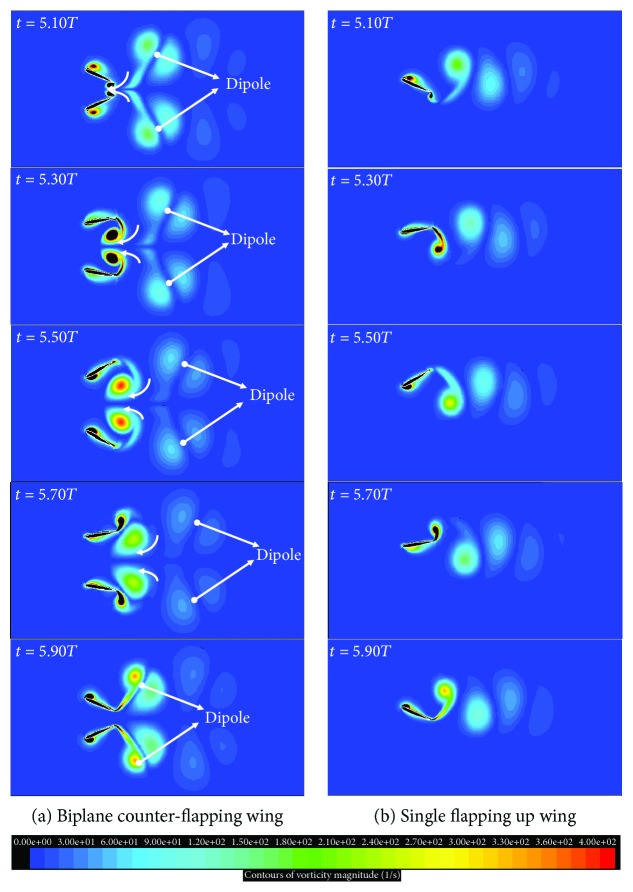
The vortex contours of the biplane counter-flapping wing and single flapping up wing during a flapping cycle.

**Figure 9:**
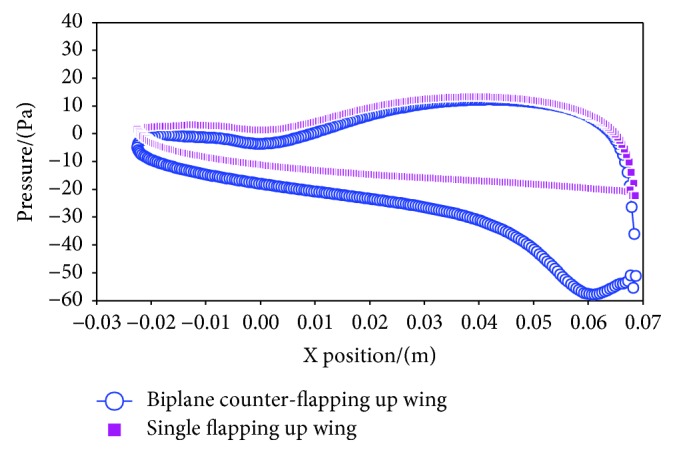
The pressure of the up and down surfaces of the counter-flapping wing and single flapping up wing at *t* = 5.10 *T*.

Figure 8 presents the vortex contours of the two considered wings in the above section. Obviously, two dipoles which have two pairs of leading and trailing edge vortices of opposite rotation are observed in the wake of the biplane counter-flapping wing; however, only single row vortex street is observed for the single flapping up wing, and the dipole moves downstream carrying momentum with it to generate larger thrust of the wing, which is the reason why the biplane counter-flapping wing has better thrust-generating performance than the single flapping wing. According to the study on the insect flapping wing by Wang [16], she also concluded that the dipole can enhance the aerodynamic force-generating performance of the wing, which is consistent with the conclusion indicated in this paper. It is also found in this figure that the vortices of the biplane counter-flapping wing have stronger strength than the single flapping up wing due to the up and low wing interaction, which will lead that the wing has larger pressure gradient between up and down surfaces of the wing as shown in Figure 9 and resulting the wing to generate larger thrust.

### 4.2. Mean Wing Spacing Influence Study

To study the effect of mean wing spacing *y*_*s*_ on the propulsive performance of the biplane counter-flapping wing, we investigate another three different mean wing spacings *y*_*s*_ = 1.40*c*, 1.75*c*, and 2.00*c*. For each of them, *ϕ* = −120° to 60° (with interval of 30°) where the biplane counter-flapping wing has positive mean thrust coefficient and propulsive efficiency as shown in Figure 6 is considered.

**Figure 10:**
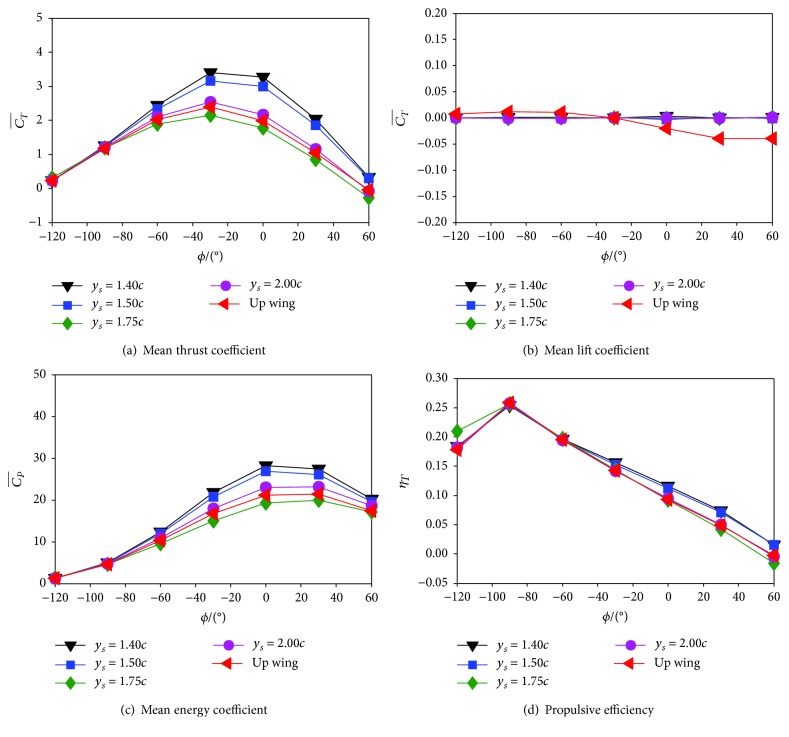
The performance of the biplane counter-flapping wing with different mean wing spacing *y*_*s*_.

Figure 10 shows the performance of the biplane counter-flapping wing with different mean wing spacing *y*_*s*_. Note that the results of the biplane counter-flapping wing with *y*_*s*_ = 1.50*c* and single flapping up wing are also presented for comparison.

It is clear in Figure 10(a) that similar variation trend of the mean thrust coefficient of the wing with phase angle is observed; however, the biplane counter-flapping wing with *y*_*s*_ = 1.40*c* has the largest amplitude, and the biplane counter-flapping wing with *y*_*s*_ = 1.75*c* has the smallest mean thrust coefficient amplitude, when the wing with phase angle *ϕ* = −60° to 60°. On the other hand, when the wing with phase angle *ϕ* = −120° to −90°, the considered wing almost has identical amplitude. These results indicate that there exists a worse mean wing spacing, where the biplane counter-flapping wing generates the least thrust.

It is obvious in Figure 10(b) that the mean lift coefficient of the considered wing is all close to zero due to the biplane counter-flapping wing as we conducted in the last section.

It is seen from Figure 10(c) that the variation trend of the mean energy coefficient of the wing with phase angle is similar as the mean thrust coefficient of the wing as shown in Figure 10(a).

It is seen from Figure 10(d) that for the considered four different mean wing spacings, the maximum power efficiency of the wing is achieved when the wing with *ϕ* = −90°, and the maximum power efficiency amplitude of the wing is almost identical, which indicates that the mean wing spacing has little influence on the best power efficiency performance of the wing. It is also found in this figure that the power efficiency of the wing increases with the *y*_*s*_ decreasing, when the wing with phase angle *ϕ* = −60° to 60°, except for the wing with *y*_*s*_ = 1.75*c* which has the smallest efficiency.

In order to analyze the mechanism of how the mean wing spacing influences the propulsive performance of the flapping wing details, two typically biplane counter-flapping wing cases with *y*_*s*_ = 1.50*c* and 1.75*c*, *ϕ* = 60° which have maximum propulsive efficiency difference for the studied wing with different mean wing spacing as shown in Figure 10, are analyzed in detail.

**Figure 11:**
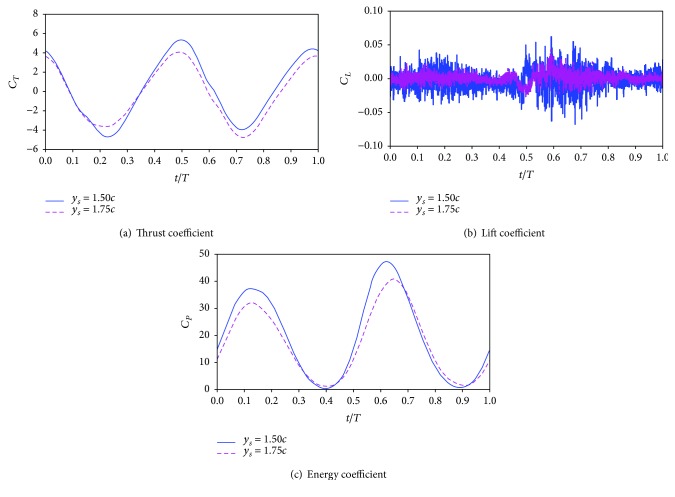
Time variation of the thrust coefficient, lift coefficient, and energy coefficient of the biplane counter-flapping wing with *y*_*s*_ = 1.50*c* and 1.75*c*, *ϕ* = 60°.

Figure 11 plots the time variation of thrust coefficient, lift coefficient, and energy coefficient of the two considered wings in this section. It is obvious in Figure 11(a) that the biplane counter-flapping wing with *y*_*s*_ = 1.50*c* has larger thrust coefficient than the wing with *y*_*s*_ = 1.75*c* almost during the whole flapping cycle, expect at *t* = 0.18 *T*–0.35 *T*, which results the wing with *y*_*s*_ = 1.50*c* to have larger mean thrust coefficient as shown in Figure 10(a). It is seen from Figure 11(b) that the lift coefficients of the two studied wings are close to zero during the whole flapping cycle; however, the wing with *y*_*s*_ = 1.50*c* has larger amplitude than the wing with *y*_*s*_ = 1.75*c*. As shown in Figure 11(c), the energy coefficient of the biplane counter-flapping wing with *y*_*s*_ = 1.50*c* has larger amplitude than the wing with *y*_*s*_ = 1.75*c* almost during the whole flapping cycle, expect at *t* = 0.70 *T*–0.90 *T*, which results the biplane counter-flapping wing with *y*_*s*_ = 1.50*c* to have larger mean energy coefficient as shown in Figure 10(c).

**Figure 12:**
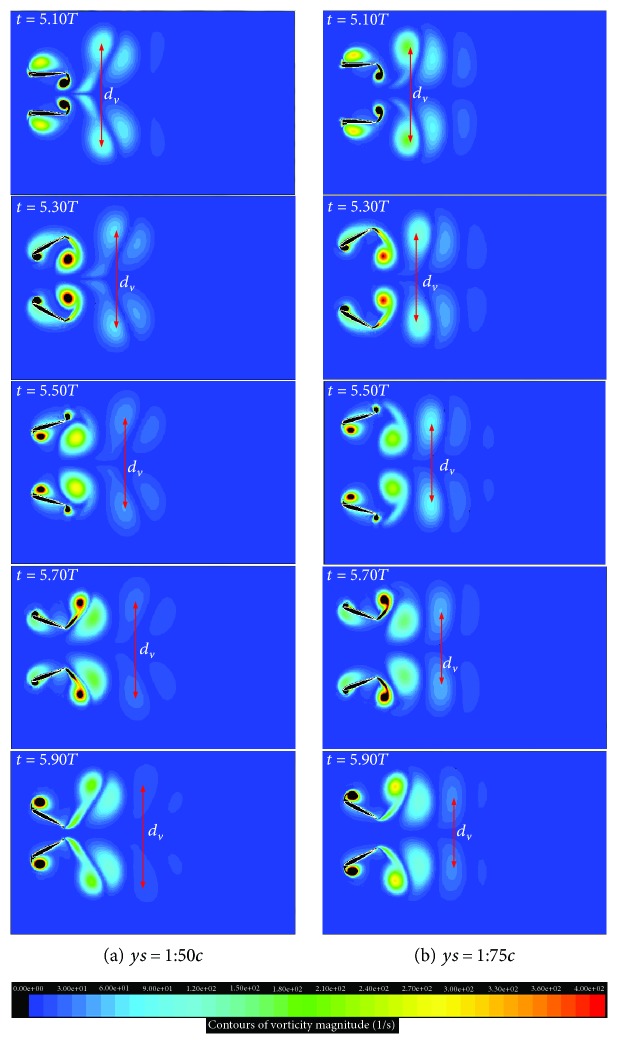
The vortex contours of the biplane counter-flapping wing with *y*_*s*_ = 1.50*c* and 1.75*c*, *ϕ* = 60° during a flapping cycle.

**Figure 13:**
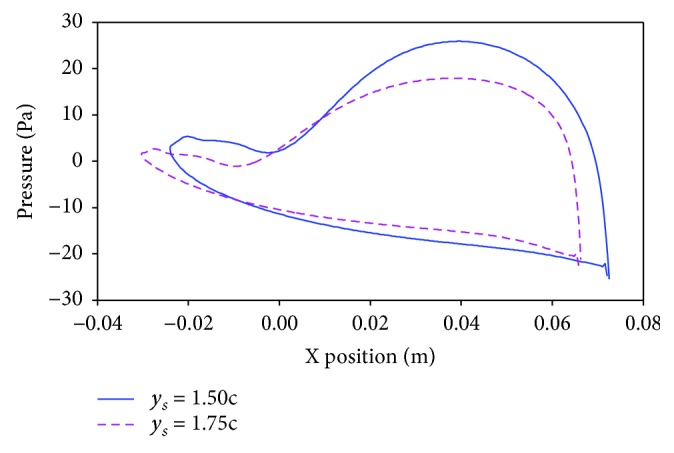
The pressure of the up and down surfaces of the counter-flapping up wing with *y*_*s*_ = 1.50*c* and 1.75*c* at *t* = 5.50 *T*.

Figure 12 shows the vortex contours of the two considered wings in this section. Obviously, similar vortices are observed around the two studied wings; however, the biplane counter-flapping wing with *y*_*s*_ = 1.50*c* has stronger vortices than the wing with *y*_*s*_ = 1.75*c*, which will lead the wing that has larger pressure gradient between up and down surfaces of the wing as shown in Figure 13 and resulting the wing to generate larger thrust. Moreover, the biplane counter-flapping wing with *y*_*s*_ = 1.50*c* has larger vertical distance (as shown *d*_*v*_ in this figure) of vortices in the wake than the wing *y*_*s*_ = 1.75*c*, which also results the wing to have better propulsive performance, as conducted by Young [17] and Zhu and Zhou [18].

## 5. Conclusion

In this paper, a numerical experiment is carried out to investigate the effect of phase angle as well as the mean wing spacing on the aerodynamics of flapping wings in biplane configuration, where the incompressible Navier-Stokes (N-S) equations coupled with biplane counter-flapping motion of the wing are solved. The flow field, thrust forces, and propulsive efficiency of the wing are analyzed, and the results show that the phase angle and mean wing spacing influence on the propulsive characteristics of the biplane counter-flapping wing greatly.

With regard to the biplane counter-flapping wing with different phase angle *ϕ*, we found that the maximum mean thrust coefficient is achieved when the wing with *ϕ* = −30°, while the maximum power efficiency is achieved when the wing with *ϕ* = −90°, and it is also found that the thrust generated by the biplane counter-flapping wing has larger mean values and propulsive efficiency than the isolated single flapping wing when the phase angle of the wing is at *ϕ* = [−30°, 60°], and the maximum thrust increasing is 77.14% which is achieved at *ϕ* = 0°, and the maximum propulsive efficiency increasing is 45.96% which is achieved at *ϕ* = 30°. Due to the wing-wing interaction, the dipole and stronger vortices are observed for the biplane counter-flapping wing, therefore leading to have better propulsive performance than the isolated single flapping wing.

With regard to the biplane counter-flapping wing with different mean wing spacing *y*_*s*_, we found that there exists a worse mean wing spacing *y*_*s*_ = 1.75*c*, where the biplane counter-flapping wing generates least thrust. On the other hand, if the wing has appropriate mean wing spacing (*y*_*s*_ = 1.50*c*), the interaction of the up and low wings can lead the wing to produce larger mean thrust and propulsive efficiency; however, the maximum power efficiency of the wing is almost not changed with the variation of the *y*_*s*_. The mean wing spacing can influence the strength and vertical distance of vortices. The stronger and larger vertical distance of vortices is observed for the biplane counter-flapping wing with appropriate mean wing spacing; this is the reason for the wing to have better propulsive performance.

## Data Availability

No data were used to support this study.
